# MtDNA deletions and aging

**DOI:** 10.3389/fragi.2024.1359638

**Published:** 2024-02-15

**Authors:** Charlotte Sprason, Trudy Tucker, David Clancy

**Affiliations:** Biomedical and Life Sciences, Lancaster University, Lancaster, United Kingdom

**Keywords:** aging, mitochondria, mitochondrial DNA deletions, mitochondrial DNA, mitochondrial dysfunction

## Abstract

Aging is the major risk factor in most of the leading causes of mortality worldwide, yet its fundamental causes mostly remain unclear. One of the clear hallmarks of aging is mitochondrial dysfunction. Mitochondria are best known for their roles in cellular energy generation, but they are also critical biosynthetic and signaling organelles. They also undergo multiple changes with organismal age, including increased genetic errors in their independent, circular genome. A key group of studies looking at mice with increased mtDNA mutations showed that premature aging phenotypes correlated with increased deletions but not point mutations. This generated an interest in mitochondrial deletions as a potential fundamental cause of aging. However, subsequent studies in different models have yielded diverse results. This review summarizes the research on mitochondrial deletions in various organisms to understand their possible roles in causing aging while identifying the key complications in quantifying deletions across all models.

## 1 Introduction

The difficulty of studying aging begins with defining it because of the variety of factors it can be considered in, including chronological, behavioral, social, physiological, cellular, and molecular changes. While there is no universally accepted definition of aging, in the context of biogerontology, it can broadly be defined as the lifelong continuous loss of physiological homeostasis resulting in a continually increasing probability of pathology and death. Currently, there are 12 proposed hallmarks of aging that manifest with age, accelerate aging when experimentally accentuated, and, to varying degrees, decelerate, stop, or slow aging when targeted with therapeutic interventions (López-Otín et al., 2023). Given the non-paradigm nature of the hallmarks of aging that describe its features rather than definitive causes, biogerontology still has a way to go to understand the mechanistic underpinnings of aging, but the hallmarks do provide a reasonable basis for some potential causes (Gems and De Magalhães, 2021). While the exact nature of aging is unknown, the universality of the aging phenotype across and within species, especially in eukaryotes, suggests that an equally universal mechanism or mechanisms govern it (Shokolenko et al., 2014). This brings us to discuss two hallmarks of aging: mitochondrial dysfunction and genomic instability.

**FIGURE 1:**
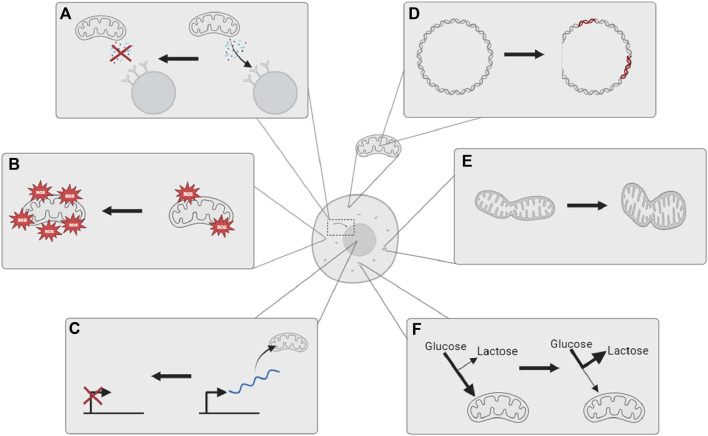
Mitochondria are observed to undergo multiple changes with age. **(A)** Reduced retrograde signaling from mitochondria to the nucleus; **(B)** an increase in oxidative stress and damage; **(C)** decreased expression of mitochondrial biogenic genes in the nucleus; **(D)** increased mitochondrial DNA mutations and deletions; **(E)** altered mitochondrial dynamics; and **(F)** metabolic shift toward glycolysis and extra-mitochondrial energy metabolism. The illustration is created using BioRender.

Mitochondria are eukaryotic organelles whose primary role is to generate adenosine triphosphate (ATP) from oxygen and dietary derivatives through oxidative phosphorylation (OXPHOS) using electron transport chain (ETC) complexes. ATPs are molecules that contain high-energy phosphate bonds and are necessary for eukaryotic cells in metabolic processes (Dabravolski et al., 2022). Mitochondria are also involved in regulating whole-cell homeostasis, providing intermediates for biosynthesis, and have roles in cell signalling, including stimulating apoptosis. All such functions of mitochondria have been observed to change with age, leading to mitochondrial and metabolic dysfunction (Figure 1).

This review explores the factors that complicate the role of mitochondrial deletions in a universal natural aging process, the current arguments made for their involvement or otherwise, and the need for future research.

## 2 Mitochondria and the mitochondrial genome

**FIGURE 2:**
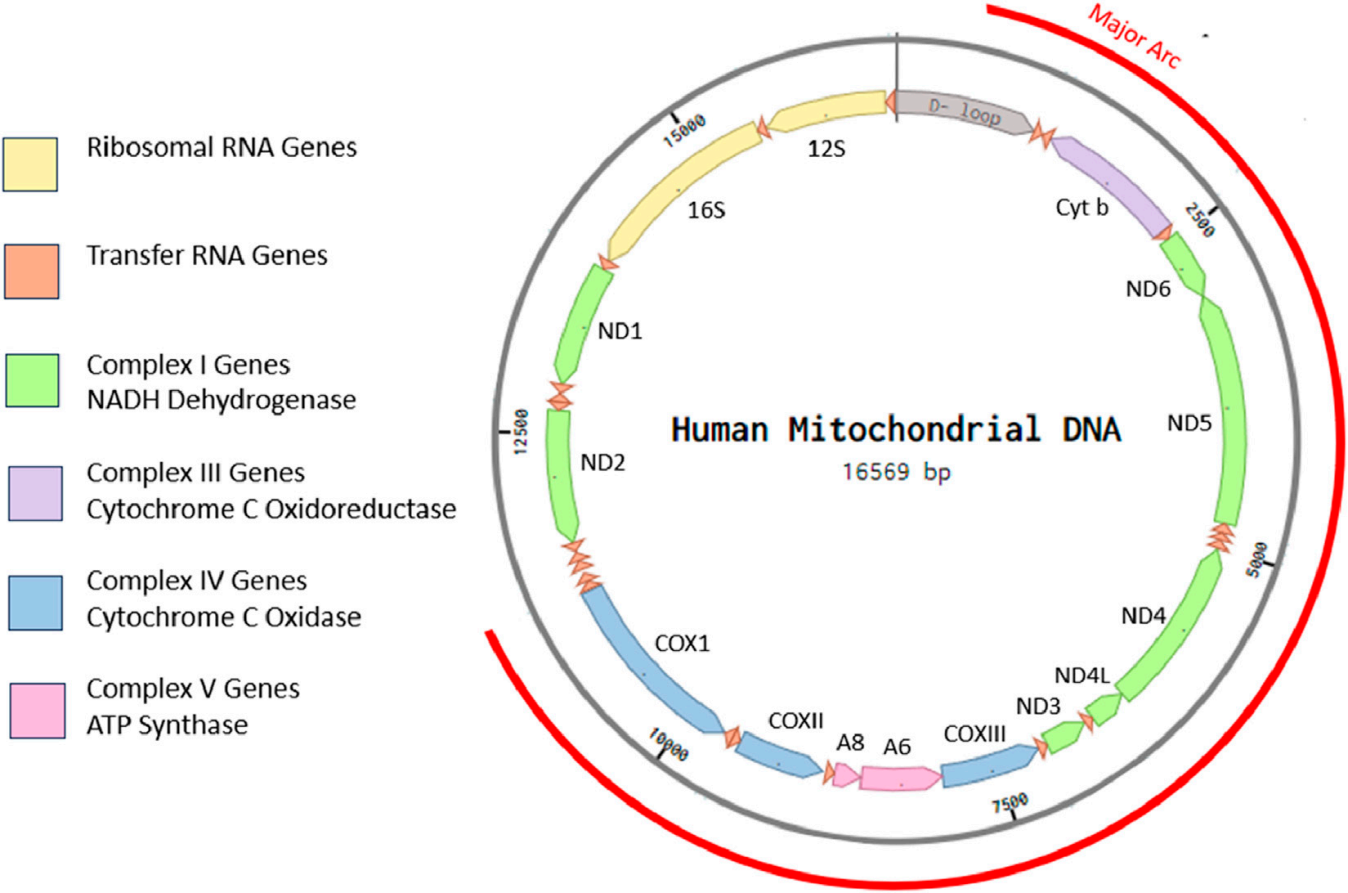
Eukaryotic cells possess multiple mitochondria, each with their own independent, vestigial, circular genome. Human mtDNA is ∼16.5-kbp long, circular, and double stranded. It contains a displacement loop (D-loop) and 37 genes coding for 22 tRNAs, 2 rRNAs, and 13 peptides with no introns. The illustration is created in Benchling from human mitochondrial DNA sequence accession: NC_012920.

Individual cells contain hundreds to thousands of mitochondria depending on their specialization and energy needs. As former bacterial endosymbionts, every mitochondrion possesses a short genome independent of the nucleus (Chocron et al., 2019). Most mitochondrial proteins are now coded for in the nuclear genome, but mitochondria retain some of their own genome, known as mitochondrial DNA (mtDNA), a molecule that is relatively conserved across the animal kingdom in terms of structure and gene organization (Gissi et al., 2008). In addition to independently replicating their own genome, these genes encode for many of the essential components of the ETC and OXPHOS structures and are, thus, crucial for ATP generation and cellular function (Anderson et al., 1981; Stewart and Chinnery, 2021). The human mitochondrial genome is depicted in Figure 2.

### 2.1 mtDNA inheritance

**FIGURE 3:**
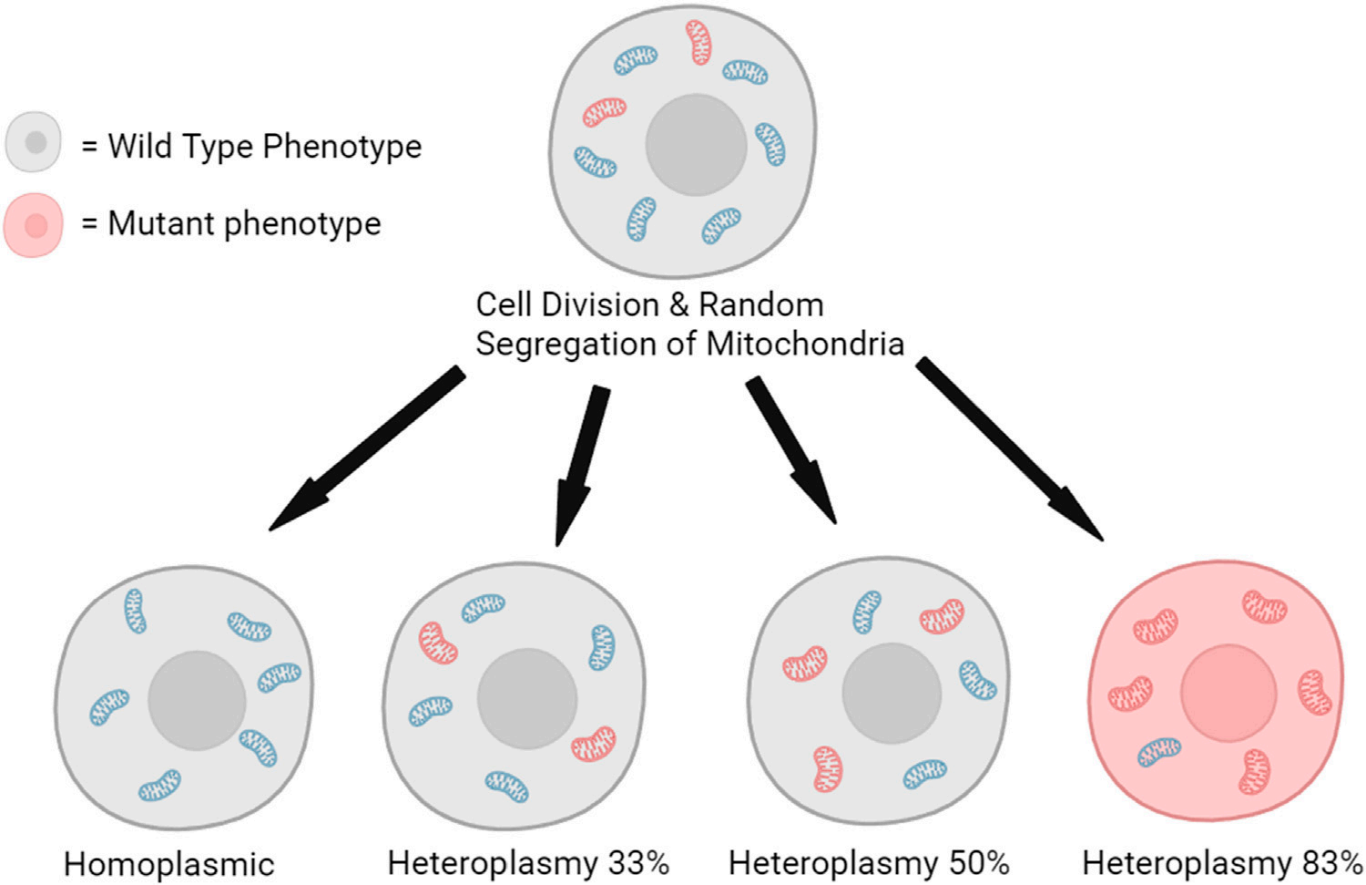
Random segregation of mitochondria during cell division can lead to the clonal expansion of mutated mitochondrial DNA and the production of a minority of cells with a mutant phenotype (Kauppila et al., 2018). The illustration is created using BioRender.

In sexually reproducing species, mtDNA is exclusively maternally inherited while paternal mtDNA is eliminated by various mechanisms within the egg post-fertilization (Fan et al., 2008; Sato and Sato, 2013; Rojansky et al., 2016; Lee et al., 2023). Through poorly understood mechanisms, some maternal mtDNA is also selected against during fertilization (Latorre-Pellicer et al., 2019). As a result, embryos have largely homoplasmic mtDNA (Fan et al., 2008), but through development and age, spontaneous mtDNA mutations can accumulate and dissipate, leading to different levels of mitochondrial heteroplasmy between cells (Figure 3). Clonal expansion of some mtDNA variants or mutations can have a deleterious effect, leading to diseased phenotypes once the mutant mitochondria frequency exceeds a harmful threshold within sufficient cells (Wallace and Chalkia, 2013).

In a phenomenon known as mother’s curse, it is theorized that the near-exclusive maternal inheritance of mtDNA reduces the ability of natural selection to select against mtDNA mutations that have a deleterious effect on males if they have a neutral, beneficial, or only mildly deleterious effect on females (Gemmell et al., 2004). When comparing 13 *D. melanogaster* lines with isogenic nuclear genomes and different mtDNA haplotypes, the effects of mtDNA haplotypes on the metabolic rate were more prominent in males than females of each haplotype (Camus et al., 2012). In another study, it was shown that the effects of mtDNA haplotypes on the metabolic rate were more prominent in males than females and male-specific negative correlations were observed across haplotypes for longevity and metabolic rate. These effects on longevity were also amplified by stressful environments (Nagarajan-Radha et al., 2020). Regardless of the total contribution that maternal inheritance of mtDNA has on life-history trait evolution, these studies demonstrate the interconnected and pleiotropic effects of mtDNA mutants on longevity from genetic and environmental influences.

In species where sex is determined by either homogametic or heterogametic sex chromosomes, it has been frequently observed that the homogametic sex lives longer (Austad and Fischer, 2016). This trend includes longer-lived female mammals (XX), male birds (ZZ), and various insects that utilize either male or female heterogamety. In a quantification of the differences in lifespan between the sexes of 229 species, it was found that homogametic females live, on average, 20.9% longer than their male counterparts, whereas homogametic males only live 7.1% longer than their heterogametic female counterparts (Xirocostas et al., 2020). This comparatively increased rate of aging in males could be partly attributed to the mother’s curse, but because male homogametic individuals still live longer on average than heterogametic females, it is also demonstrative that there are other genetic factors that influence the natural rate of aging more than the prospective maternal inheritance of deleterious mtDNA. Regardless, with a plethora of evidence both for and against mother’s curse as a concept and the effect of environment on phenotypes, it is difficult to refute mother’s curse, and it is even more difficult to refute the contribution of mtDNA and mitochondrial activity to aging.

### 2.2 mtDNA mutations

Because mtDNA is almost entirely coding, mitochondrial mutations frequently disrupt the genes essential for mitochondrial function. The ratio of nuclear DNA mutations to mtDNA mutations differs between the tissues of the same organism as broadly as it does between species (Lax et al., 2011; Allio et al., 2017), but in animals, mtDNA always has a faster natural rate of mutation, making it prone to deleterious mutations and pathology. This rate of mutation is attributed to a variety of factors: mtDNA has a comparatively more error-prone replication and limited error-repair machinery, it is condensed by nucleoprotein complexes instead of histones, and it resides at the inner mitochondrial membrane where reactive oxygen species (ROS) are produced, making it susceptible to oxidative damage (Bogenhagen, 2012; Chevigny et al., 2020). mtDNA mutations are thought to arise due to errors during replication and repair due to unrepaired DNA damage, such as base misincorporation events (Sanchez-Contreras and Kennedy, 2022).

The consequences of mtDNA mutations vary based on the time of their occurrence within the organism’s lifespan, tissue type, and the total mtDNA content of the cells (Wallace and Chalkia, 2013; Stewart and Chinnery, 2015; Filograna et al., 2019). Because cells have several copies of mitochondria, pathologies only occur when deleterious mutations reach a certain threshold of mutations per mitochondrion and/or sufficiently dysfunctional mitochondria per cell, though this threshold does not necessarily reflect the proportion of mitochondria and may instead reflect the absolute quantity of mitochondria (Jeppesen et al., 2006; Jiang et al., 2017). This can be seen in inherited genetic conditions and has been implicated in normal aging and age-related pathologies, such as Parkinson’s disease (Corral-Debrinski et al., 1993; Melov et al., 1997; Melova et al., 1999; Lin et al., 2002; Bender et al., 2006; Kraytsberg et al., 2006).

Generally, mtDNA mutations include single-nucleotide point mutations and single base to several kilobase deletions. A key study in mice found that even when point mutations were amplified 500-fold above the wild-type (WT) levels, mice health and lifespans were unaffected (Vermulst et al., 2007). A later assay by the same group with human brain tissue concluded that mtDNA point mutations were orders of magnitude too low to account for the necessary oxidative damage to lead to the phenotypes of aging (Vermulst et al., 2008). It has since been largely agreed that mtDNA point mutations are unlikely to play a major role in aging, but the discussion on whether mtDNA deletions affect aging is still ongoing.

### 2.3 mtDNA deletions

Multiple studies have found that a majority of mtDNA deletions occur within the major arc region of mtDNA (Yui et al., 2003; Bua et al., 2006; Chen et al., 2019). The major arc contains largely identical genes across species, including human mtDNA, as depicted in Figure 1 *.* While the exact mechanism of deletions is not known, nucleic acid motifs such as repeats and secondary structures are associated with deletion formation in humans, nematodes, and rats, among other animals; therefore, it is expected that mtDNA deletions occur through a mechanism involving mtDNA repeat sequences (Melov et al., 1994; Bua et al., 2006; Yui and Matsuura, 2006). The resulting secondary DNA structures likely impact replication or repair (Fontana and Gahlon, 2020). More broadly, comparisons of mammalian species show the abundance of direct nucleotide repeats in mtDNA to be negatively associated with longevity, and the size of direct repeats has been found to correspond with more severe pathology (Khaidakov et al., 2006).

The results of mitochondrial heteroplasmy complicate research into the impact of mtDNA deletions on aging. For example, a *de novo* deletion acquired with age and present with low mitochondrial copy number may have limited pathogenic effects compared to a deleterious mtDNA variant that has clonally expanded to a greater degree (Sanchez-Contreras and Kennedy, 2022). Regardless of the pathogenic effects, mathematical modeling of the clonal expansion of mtDNA mutations shows that random segregation during mitochondrial cell division alone cannot explain the experimental evidence of mtDNA deletion accumulation in both short- and long-living organisms (Kowald and Kirkwood, 2013). Different studies continue to find diverse results across various models, along with differences in the deletion accumulation between tissues, the influence of mitochondrial copy number, disease states, and the use of different methodologies.

## 3 Mitochondrial deletions in aging

### 3.1 Mitochondrial deletions and human pathology

It has long been observed in humans that mtDNA deletions increase in some tissues with age, and they have been associated with various diseases. Neurodegenerative disorders are a leading global cause of premature death, and age is the primary risk factor for many of these (Dattani et al., 2023). Alzheimer’s patients (<75 y/o) have been shown to have 15 times more mtDNA deletions in multiple brain regions compared to age-matched control individuals (Corral-Debrinski et al., 1994). A study looking at the size of mtDNA deletions within substantia nigra neurons found that up to 52% of mtDNA was deleted in Parkinson’s patients’ neurons compared to 43% in age-matched controls (Bender et al., 2006). A more recent assay using ultra-deep sequencing of major arc mtDNA deletions from 17 individual substantia nigra neurons from two individuals with Parkinson’s disease showed with much higher sensitivity a higher number of deleted mtDNA species per neuron. By demonstrating differences in heteroplasmy between deleted mtDNA subpopulations and the rest of the neuronal mtDNA subpopulation, this confirmed the notions of the clonal expansion of somatic deletions (Nido et al., 2018). These studies suggest a link between the increased incidence and degree of mtDNA deletions and age-associated neurodegenerative diseases.

Other tissues have also shown increased mtDNA deletion incidence and size and mitochondrial dysfunction with age. A study looking at muscle fibers of deceased humans found that ETC abnormalities increased from 6% to 31% of fibers from ages 49–92 years. In the fibers with abnormal ETC, long-range polymerase chain reaction (PCR) found that some mtDNA molecules had up to 90% of their length deleted and had no full-length mtDNA genomes (Bua et al., 2006). In coronary artery disease patients, deletions within certain mitochondrial genes are thought to impact the energy metabolism of atrial appendage tissues, contributing to cardiac dysfunction (Matam et al., 2014). The common mtDNA^4977^ deletion was also found in twice as many patients with diabetes or impaired glucose tolerance compared to healthy age-matched individuals (Liang et al., 1997).

While the frequency of mtDNA deletions differs between human tissues, the most frequent type of deletion also varies. A next-generation sequencing (NGS) study of the putamen of aged volunteers found the common mtDNA^4977^ deletion to be the most abundant (Williams et al., 2013), and an NGS study of skeletal muscle samples found greater numbers of other major arc deletions (Lujan et al., 2020). The overall most frequent deletion type in humans is the common 4977 mtDNA deletion (mtDNA^4977^), which is flanked by 13 bp nucleotide repeat sequences, termed the “common” nucleotide repeat. mtDNA^4977^ has been found to accumulate in a variety of tissues during aging and may be a potential biomarker for oxidative damage (Simonetti et al., 1992; Arai et al., 2003; Vecoli et al., 2020).

One study investigated the frequency of the common mtDNA^4977^ deletion in various sections of the human brain from the ages of 67 to >80 years. The putamen samples expressed the greatest variation, from 0.16%–1% of mtDNA molecules in 67–77 y/o individuals to up to 12% of mtDNA molecules in >80 y/o individuals. Over the same age ranges, the cortex experienced a lower increase in mtDNA^4977^ frequency, ranging from 0.023%–1.2% up to 3.4%. Comparatively, the cerebellum showed a negligible change in mtDNA^4977^ frequency (Corral-Debrinski et al., 1992).

The presence of the common nucleotide repeat has been associated with longevity within some human populations. The D4a mtDNA haplotype is particularly enriched among Japanese centenarian (Alexe et al., 2007) and semi-supercentenarian populations (Bilal et al., 2008), implicating a slower rate of aging compared to the general population. This haplogroup was found to have a point mutation within the 5’ common nucleotide repeat, which borders the mtDNA^4977^ common deletion (Mikhailova et al., 2019).

A second haplotype, the N1b mtDNA haplotype, expresses a different point mutation disruption in the 5’ common nucleotide repeat. A study using deletion breakpoint distributions compared the frequency of deletions in the frontal cortex tissue from individuals with the N1b haplotype to age-matched controls. It was found that the frequency of all deletions was decreased while the ratio of its frequency compared to other deletions was retained (Guo et al., 2010). These studies could suggest that mtDNA deletion formation can be reduced by disrupting the common nucleotide repeat. If this is true, then it is possible that the D4a mtDNA haplotype works in a similar manner: the disruption of the common repeat decreases the likelihood of developing corresponding deletions over a lifetime, thus decreasing the rate of aging and increasing longevity. This is, however, yet to be explicitly demonstrated. Interestingly, given that the ratio of deletion types had remained consistent between N1b and the control mtDNA, it may suggest that the common nucleotide repeat may be involved in the formation of multiple types of deletions through a more complicated mechanism rather than simply the theories concerning localized repeat sequences. Alternatively, the common nucleotide repeat and mutations within it may have a greater pleiotropic role in cellular health and organismal longevity other than mtDNA deletion formation. While suggestive of the connection between mtDNA deletions and aging, these studies cannot demonstrate a definitive link between the two.

Overall, studies on human mtDNA deletions have so far been limited. These studies typically demonstrate the correlation between mtDNA deletions and pathologies of aging but provide no causative link. Most also rely on methods that only amplify the common mtDNA^4977^ deletion or its associated breakpoints. Despite being the most abundant deletion between mtDNA molecules, mtDNA^4977^ often only makes up to 10% of the total deletions, even in tissues which have a high common deletion load (Kraytsberg et al., 2006). Additionally, the methodologies used frequently investigate to what degree mtDNA is lost between a few mtDNA samples rather than how frequently different mtDNA deletions occur across cells or tissues. Because of this, the effects of the total deletion load are often not considered in these studies.

The participants included in these studies also limit research. There is a particular focus on age groups over 50 years, even though intrinsic mortality begins to increase from 25 years of age. Investigating older participants also increases the chance that undiagnosed pathologies of aging may obscure the results of the control groups. Human participants often also have very different diets and lifestyles, which induce great variability between trials, even when comparing age-matched individuals. Using animal models to investigate mtDNA deletions resolves several of these issues since deletions can be induced, lifestyles can be controlled, all tissues can be evaluated within the populations at different time points, and lifespans can be observed along with the fitness parameters throughout the said lifespan.

Despite their flaws, it is important to acknowledge that studies in human subjects demonstrate that mtDNA deletion accumulation occurs within certain tissues with age and that there is a correlation between them and the aging process in humans, and even if a causative link cannot be established, these studies are immensely important in understanding human aging.

### 3.2 Mutator mice and the origin of the mitochondrial DNA deletion hypothesis

Mitochondrial deletions as a potential cause of aging gained a degree of interest after several key studies in the early 2000s that utilized polymerase γ (POLG) mutator mice. POLG is a nuclear-encoded mtDNA polymerase; it has been observed that defective POLG in humans leads to increased mtDNA deletions and premature aging hallmarks, such as parkinsonism and early menopause (Luoma et al., 2004). A murine study used a knock-in method to perform a single base substitution to produce homozygous and heterozygous mice with proofreading-deficient POLG. Using a cloning and sequencing PCR method, they found short 12 kb mtDNA fragments in the hearts and brains of aged mice. POLG^mut/mut^ mice had reduced lifespans but, more notably, expressed characteristically human premature aging phenotypes, such as hair loss and graying, weight loss, and spinal curvature. Wild-type and POLG^mut/+^ mice exhibited no abnormal phenotypes, and their tissue samples contained fewer deletion fragments (Trifunovic et al., 2004b).

A separate research group later reevaluated POLG mutator mice using a random mutation capture PCR method to assess the frequency of mtDNA point mutations and deletions in the hearts and brains of both young and old mutator mice. POLG^mut/mut^ and POLG^mut/+^ mice showed levels of point mutations around 100 times greater than those of wild-type mice, but the number of large mtDNA deletions in aged POLG^mut/mut^ mice was around 150-fold greater than that in young wild-type mice compared to only a 25-fold increase in POLG^mut/+^ mice (Vermulst et al., 2008). Because it was only the POLG^mut/mut^ mice that had premature aging phenotypes and decreased longevity, these results suggested that mtDNA deletions, and not mtDNA point mutations, were responsible for the accelerated aging.

Mutator mice investigations provided some of the first causative links between mtDNA deletions and aging, leading to a surge of interest in the mtDNA deletion hypothesis of aging. This circular damage theory suggests that exposure to extrinsic and intrinsic DNA-damaging reagents combined with reduced mitochondrial DNA repair capacity leads to mtDNA deletions, which, in turn, cause mitochondrial dysfunction and increased ROS production, and this leads to further mtDNA damage. Although ROS-induced damage theories of aging are largely discredited today, there is still an ongoing debate as to whether mtDNA deletions are a major contributor to aging.

### 3.3 Proteomic opposition to the mitochondrial DNA deletion hypothesis

A selection of findings challenges the mtDNA deletion hypothesis. Given the lack of introns, large deletions would be expected to theoretically lead to the loss of tRNAs and reduced mRNA transcripts, including that of ETC components. Bulk tissue analysis, however, found no significant reduction in the RNA levels of POLG^mut/mut^ mice, and ETC components were found to be unstable with quick protein turnover (Edgar et al., 2009). The quick protein degradation instead suggests that deleterious mtDNA point mutations were in effect, not deletions. By using bulk tissue analysis, this study loses the visibility of trends, potentially overseeing *de novo* mutations that were exclusively burdening certain tissues.

Twinkle is a nuclear-encoded mtDNA helicase that forms a replisome with POLG and is essential to human mtDNA maintenance and copy number (Tyynismaa et al., 2004). In one study, a mutant Twinkle gene was knocked into a mouse model. Long-range PCR from Twinkle mutant mice samples showed mtDNA deletions up to ∼13 kB long and symptoms of ETC deficiency in various tissues, including the brain and muscle, but the mice exhibited no reduced lifespan or premature aging phenotype (Tyynismaa et al., 2005). As Twinkle mice showed similarly large mtDNA deletions to POLG mutator mice but no premature aging phenotype, it suggests that aging phenotypes observed in POLG^mut/mut^ mice could be an off-target effect of POLG dysfunction rather than simply the accumulation of mtDNA deletions. Alternatively, the premature aging phenotype could be a side effect of POLG overexpression, which may cause a loss of mtDNA copy number through its interaction with Twinkle.

### 3.4 The mtDNA copy number depletion debate

One consideration of using mutator mice models of mtDNA deletions in aging is that POLG^mut/mut^ mice show decreased mtDNA copy number. On observing mutator mice fertility phenotypes, it was found that decreasing the mtDNA copy number worsened the mitochondrial dysfunction, and increasing the copy number rescued the phenotype even when the mutation load was constant (Jiang et al., 2017). This suggests that a decreased mtDNA copy number could be a more significant contributor to aging phenotypes than mtDNA deletion frequency; however, Twinkle mutant mice experiments showed otherwise. Twinkle mutant mice exhibit a decrease in mtDNA copy number, yet they show no premature aging phenotypes (Tyynismaa et al., 2004; Tyynismaa et al., 2005). Given that Twinkle and POLG form a replisome, mutator mice copy number reduction could be a by-product of unintended Twinkle disruption by the overexpression of engineered mutant POLG. This would instead suggest that copy number reduction does not play a role in producing premature aging phenotypes.

To reduce the occurrence of mtDNA copy number depletion, *Drosophila melanogaster* were engineered to have an endogenous POLG promoter and cis-regulatory elements controlling a proofreading-deficient copy of POLG, preventing its overexpression so that mtDNA copy number remained consistent (Samstag et al., 2018). These POLG^mut/mut^ *D. melanogaster* individuals exhibited a premature aging phenotype in the form of progressive locomotor defects, loss of dopaminergic neurons, and lifespan reduction from 75 days for the wild type to 64 days. This study, thus, suggested that mtDNA copy number depletion or POLG overexpression are not the primary causes of the characteristic premature aging phenotype in POLG^mut/mut^ models, but because this study did not quantify any deletions, it can only be inferred that mutant POLG increases the incidence of mtDNA deletions and that this causes the observed premature aging phenotypes.

Together, these studies highlight the complications arising from editing genes with pleiotropic effects due to unintended consequences on cellular regulation. They demonstrate how variations in methodology from promoters on genes of interest to using different models can further exacerbate the differences in results between studies or be used to mitigate unintended consequences. They also manifest the complexities of heteroplasmy in the expression of disease phenotypes. For healthy cellular function, certain quantities of functional products translated from the mtDNA are required. Hence, when the mtDNA copy number is lost, although the degree of mtDNA heteroplasmy remains constant, diseased phenotypes can emerge. This highlights the importance of determining the exact quantities of mtDNA deletions rather than just the relative quantities of deleted and complete mtDNA molecules.

### 3.5 Mitochondrial DNA deletion relevance in the natural aging process

**FIGURE 4:**
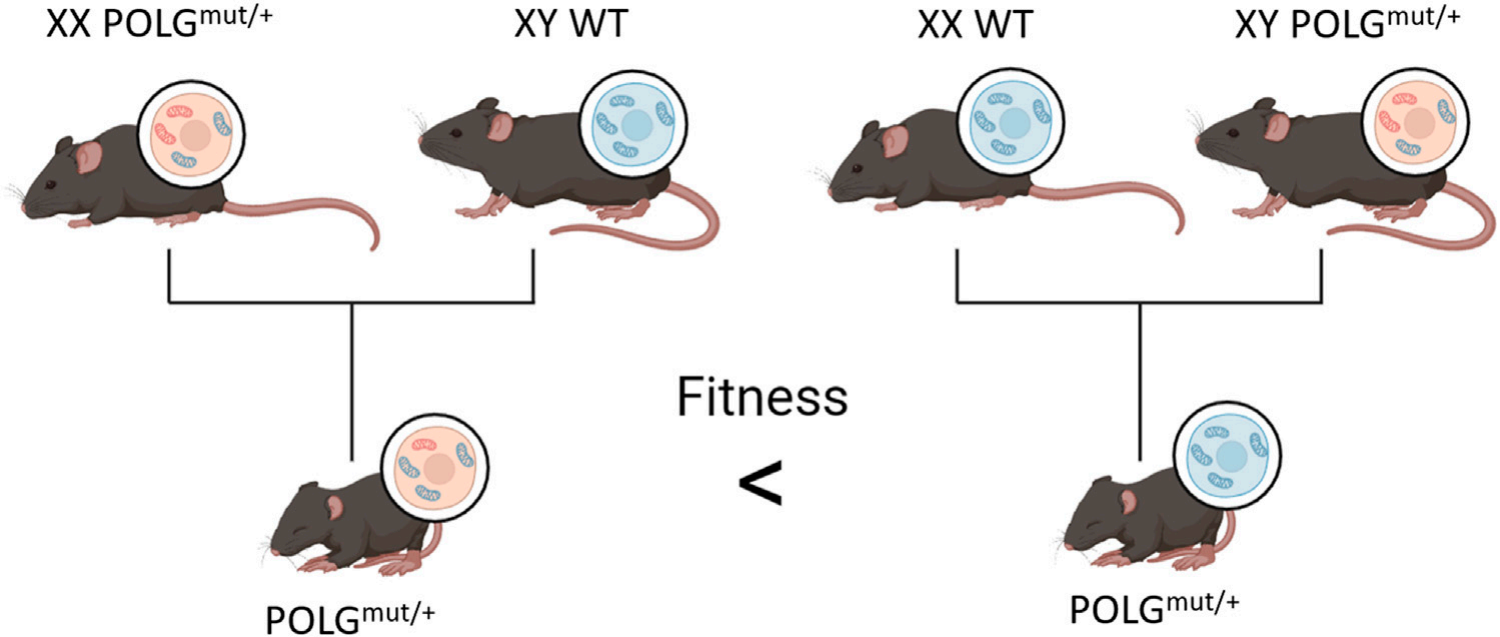
Offspring of POLGmut/+ females have decreased fitness compared to the offspring of WT females, even when all offspring carry the same chromosomal alleles. The figure is created using BioRender.

One drawback that arises from the breeding of POLG^mut/mut^ organisms by crossing heterozygotes is that regardless of their own genotypes, the offspring of female POLG^mut/mut^ mice consistently have decreased fitness and lifespan compared to the offspring of wild-type females (Figure 4) (Ross et al., 2013; Ross et al., 2014). This may be due to the clonal expansion of mtDNA mutations within the offspring acquired from the mother. Since homozygotes are bred from heterozygous parents, it can be inferred that all POLG^mut/mut^ mice start with reduced fitness and an existing mtDNA mutation load from their mother in addition to any de novo deletions they may acquire through development and age. This would indicate that mtDNA mutations are not a factor in natural aging as WT mice do not possess sufficient mutations in their germline to affect their offspring.

Random mutation capture PCR of mice brains and hearts found that mtDNA deletions increased slightly throughout the lifespan of WT and POLG^+/mut^ mice with no premature aging phenotypes, but POLG^mut/mut^ mice experienced an accelerated accumulation of a 7-to-11-fold increase over aged WT and POLG^+/mut^ levels and exhibited premature aging phenotypes across many tissues of the body (Vermulst et al., 2008). This would suggest that the mtDNA deletion threshold to cause premature aging is somewhere between 7 and 10 times greater than WT levels. This is likely too high to happen naturally outside of certain diseases, which, in turn, would suggest that mtDNA deletions are unlikely to be a cause of natural aging. Ultra-deep sequencing analysis of WT mouse livers show little change in mtDNA deletion frequency throughout WT mice lifespans (Ameur et al., 2011). Although Ameur et al. (2011) did not investigate POLG^mut/mut^ mice, it could suggest that not all tissues in POLG^mut/mut^ would accumulate mtDNA deletions like the heart and brain and, therefore, that the loss of POLG could be causing the aging phenotype instead of the resulting mtDNA deletions.

WT mouse hearts and brains appear to accumulate mtDNA mutations with age, while their livers do not (Vermulst et al., 2008; Ameur et al., 2011). A study in WT rats found the rat 4834 bp common mtDNA deletion to increase with age by two-fold in the liver and eight-fold in the brain. Interestingly, a caloric restriction diet was able to reverse the aged rats’ liver deletion load back to the levels in young adults but had no impact on the brain deletion loads (Cassano et al., 2004). The diversity between these WT murine models’ capacities to accumulate mtDNA deletions in different tissues and the ability of caloric restriction to reverse some but not all of these deletions demonstrate that the link between mtDNA deletions and aging is multifactorial and involves tissue- and species-specific processes. It should be noted that these studies used a collection of different PCR and sequencing methodologies investigating either the accumulation of many types of deletions or one type, such as the murine equivalents of the common deletion, and it could be that these differences are the cause of differing results.

In invertebrates, female *D. melanogaster* can, similarly to mice, pass existing mtDNA mutation loads to their offspring, and both sexes accumulate deletions at different frequencies between tissues. In contrast to the offspring of female POLG^mut/+^ mice, which had reduced fitness within one generation (Ross et al., 2013; Ross et al., 2014), POLG^mut/+^ the offspring of *D. melanogaster* typically express the same aging phenotype as WT flies. When the POLG mutation was propagated through generations of female POLG^mut/+^ *D. melanogaster* selection, a reduction in lifespan was only found to occur after 35 generations, therefore, it was suggested that the clonal expansion of a mother’s mtDNA deletions has minimal effect in flies (Kauppila et al., 2018). However, without investigating mtDNA deletion load, there is insufficient evidence that deletions may be inherited or might be the cause of the reduced lifespan after sufficient generations of POLG^mut/+^ flies. *D. melanogaster* may also have innate resistance/selection against mtDNA deletion accumulation as their development is arrested in the late larval stages if they carry too many mutations or too strong a deletion (Bratic et al., 2015).

*D. melanogaster* were found to show tissue-based differences in mtDNA accumulation when they are split into the head, thorax, and abdomen. Using PCR to investigate the variety of mtDNA deletions in flies, it was found that the thorax showed the strongest mitochondrial deletion signals, and these signals appeared the strongest in the old flies. These, however, were not quantified (Yui and Matsuura, 2006). In an assay where mtDNA deletions in the thorax were quantified, an increase in mtDNA deletions with age was found, but this increase was insignificant. This is suggestive that mtDNA deletions do not increase throughout the lifespan of WT *D. melanogaster* and are, therefore, unlikely to influence their natural aging. (Kauppila et al., 2018).

Haroon et al. (2018) used POLG mutant *Caenorhabditis elegans* strains to screen mitochondrial pathology pathways. By using the same random mutation capture PCR method as in the work of Vermulst et al. (2007), they were able to find elevated levels of deletions, mitochondrial dysfunction, and reduced lifespan within POLG mutants. These phenotypes could be alleviated by editing certain biological pathways, such as IGF-1 signaling pathway elements, which have been frequently manipulated in various models in association with longevity (Haroon et al., 2018). This further demonstrates the potential pleiotropic influence of POLG mutations and how they potentially reduce the lifespan indirectly through other well-known pathways rather than through mtDNA deletion accumulation.

### 3.6 A lack of consistent quantification

Large major arc genes such as MT-CO1 are more likely to be disrupted by mtDNA deletions. MT-CO1 encodes cytochrome oxidase (COX), a subunit of respiratory complex IV. Complex IV deficiency has been linked to multiple diseases and is often found in correlation with mtDNA deletions (Bender et al., 2006). If a mutated version of MT-CO1 clonally expands to a sufficient level, the cell becomes deficient in COX. Using a histochemical stain to analyze the number of COX-deficient cells is a simple and cheap method to predict mtDNA deletion frequency (Vermulst et al., 2008), but this method only confirms that the number of inactive MT-CO1 copies has exceeded a pathogenic threshold, which, as previously discussed, can be distorted by changes in the mtDNA copy number (Jiang et al., 2017). Therefore, it does not provide an accurate quantification of the frequency of the various mutations within mtDNA molecules.

Various polymerase chain reaction (PCR) methods can be used to help quantify mtDNA deletions through the amplification of target DNA. The initial 2004 mutator mice experiment used a cloning and sequencing method where PCR was used to amplify cytochrome b and a non-coding control region before they were cloned into vectors for sequencing (Trifunovic et al., 2004a). This, however, only assessed the frequency of the molecules with large deletions of cytochrome b and provided no information on the majority of the mtDNA.

**FIGURE 5:**
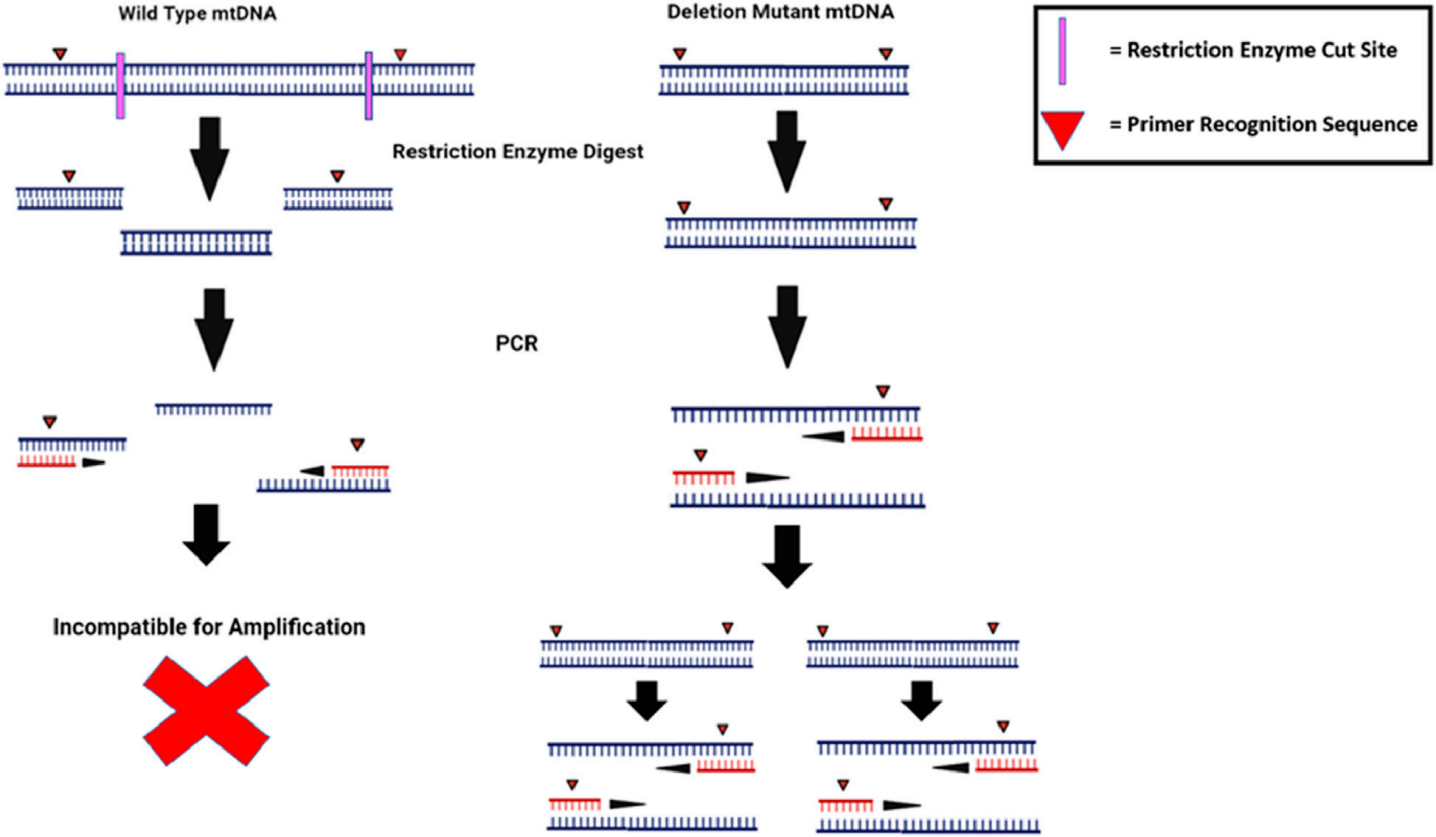
Random mutation capture methods improve the sensitivity of RT-PCR assays by exclusively amplifying rare and un-cleaved mutant DNA. RMC prevents rare *de novo* mutations from getting lost in a WT background by only amplifying mtDNA in which the specific restriction enzyme sites have been deleted. The frequency of molecules with deletions is determined by comparing the data to a PCR in which all mtDNA (WT and mutant) is amplified. The illustration is created using BioRender.

Vermulst et al. (2008) used random mutation capture (RMC) to analyze mutator mice. A restriction enzyme was used to cleave WT mtDNA before quantitative real-time PCR (qRT-PCR) amplification so that only certain mutant mtDNA would be amplified and, therefore, quantified (Figure 5). RMC found WT mutator mice to have 10 times less mtDNA mutations than previously reported by cloning and sequencing. RMC can also only identify mutants with specific deletions, but it has increased sensitivity and reduced risk of PCR mutagenesis, and it was this sensitivity to which the smaller amount of mtDNA mutations recorded was attributed (Figure 5).

In long-distance single-molecule PCR, mtDNA is diluted to a point of one template per PCR reaction. This allows for whole-molecule-scale detection of deletions. One such assay used primers that bound in the D-loop and minor arc to amplify up to 14.5 kb mtDNA. Deletion mutants could be identified as shorter mtDNA molecules that separated from longer WT in high-resolution gel electrophoresis. For this, quantification adjustment was needed to account for the effects of DNA polymerase processivity and the increased likelihood of the successful amplification of the shorter deletion mutants compared to WT mtDNA (Kraytsberg et al., 2009). Although this method takes the whole mtDNA molecule into account, the quantification is crude and provides limited information on the number and size of specific deletions.

**FIGURE 6:**
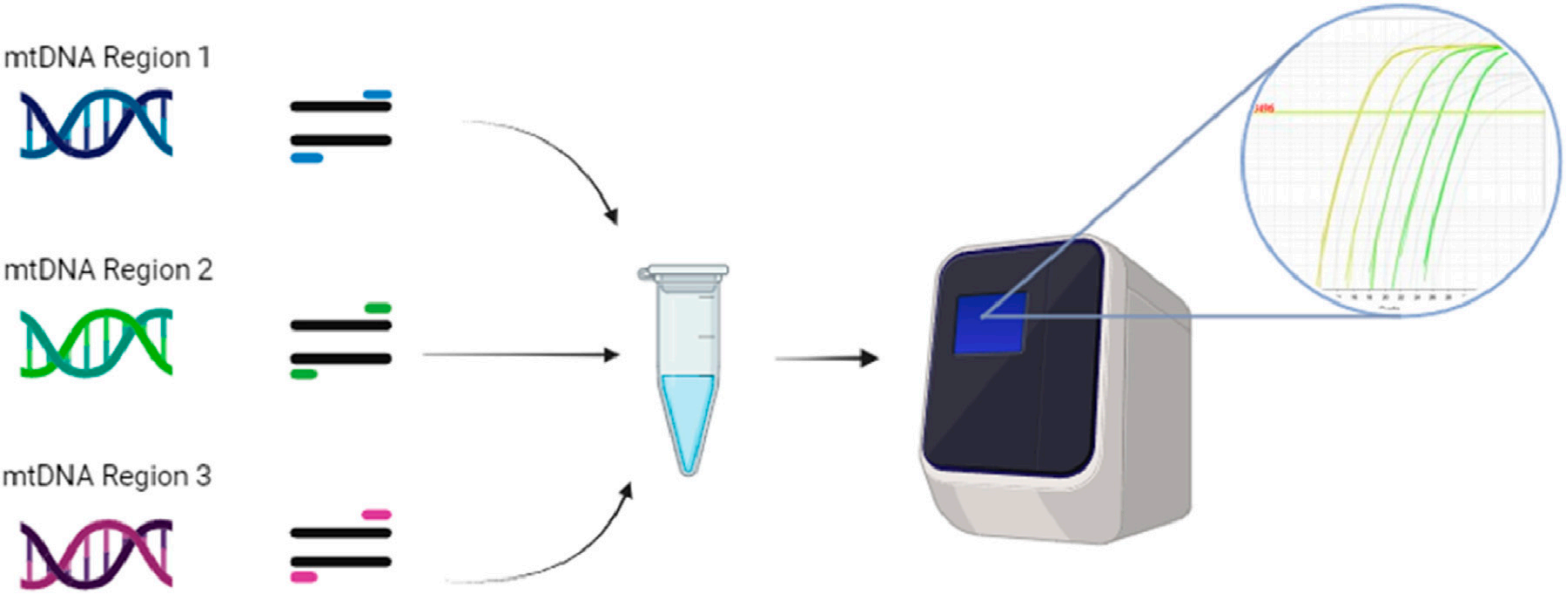
Triplex RT-PCR can combine individual mtDNA templates from the D-loop, major arc, and minor arc with their specific primers into one RT-PCR reaction. The three different templates and their primers are run through RT-PCR, where the binding of the primers’ different fluorescent probes provides unique signals, which software can identify as the WT or mutant versions of each template. The illustration is created using BioRender.

Multiplexing PCR assays can save time and reagents by amplifying different templates in a single RT-PCR reaction. Triplex RT-PCR was used to simultaneously detect mtDNA deletions over the major arc, minor arc, and D-loop (Figure 6). This reduced the issues caused by long-template DNA and provided more precise deletion loci. Quantification accuracy was also increased by using computer software over gel analysis (Rygiel et al., 2015).

Determining the varying levels of heteroplasmy between the cells of the same tissue is another quantification problem. Droplet digital PCR (ddPCR) with mutation-specific fluorescent probes was used in an assay to quantify the mtDNA heteroplasmy of individual cells, but because WT and mutant-specific probes were used, it did not provide information on the different types of deletion mutants (Maeda et al., 2020). DdPCR improves assay throughput and precision by using a water–oil emulsion to nanoliter partitions without the need for microwells or microfluidic chambers (Hindson et al., 2011).

While PCR methods can provide various forms of mtDNA deletion quantification, they are limited by factors such as DNA polymerase processivity and assay throughput and also often lack the sensitivity to quantify low-level heteroplasmies. In addition, since investigating new regions with PCR requires sequencing, breakpoint analysis, and primer optimization, most studies only analyze the common deletion or major arc genes, as a result of which rarer minor arc deletions with potentially potent outcomes are left unaccounted for. However, because they are quicker and cheaper than full sequencing, they are, thus, useful when looking for a low number of known targets (Liu et al., 2021).

**FIGURE 7:**
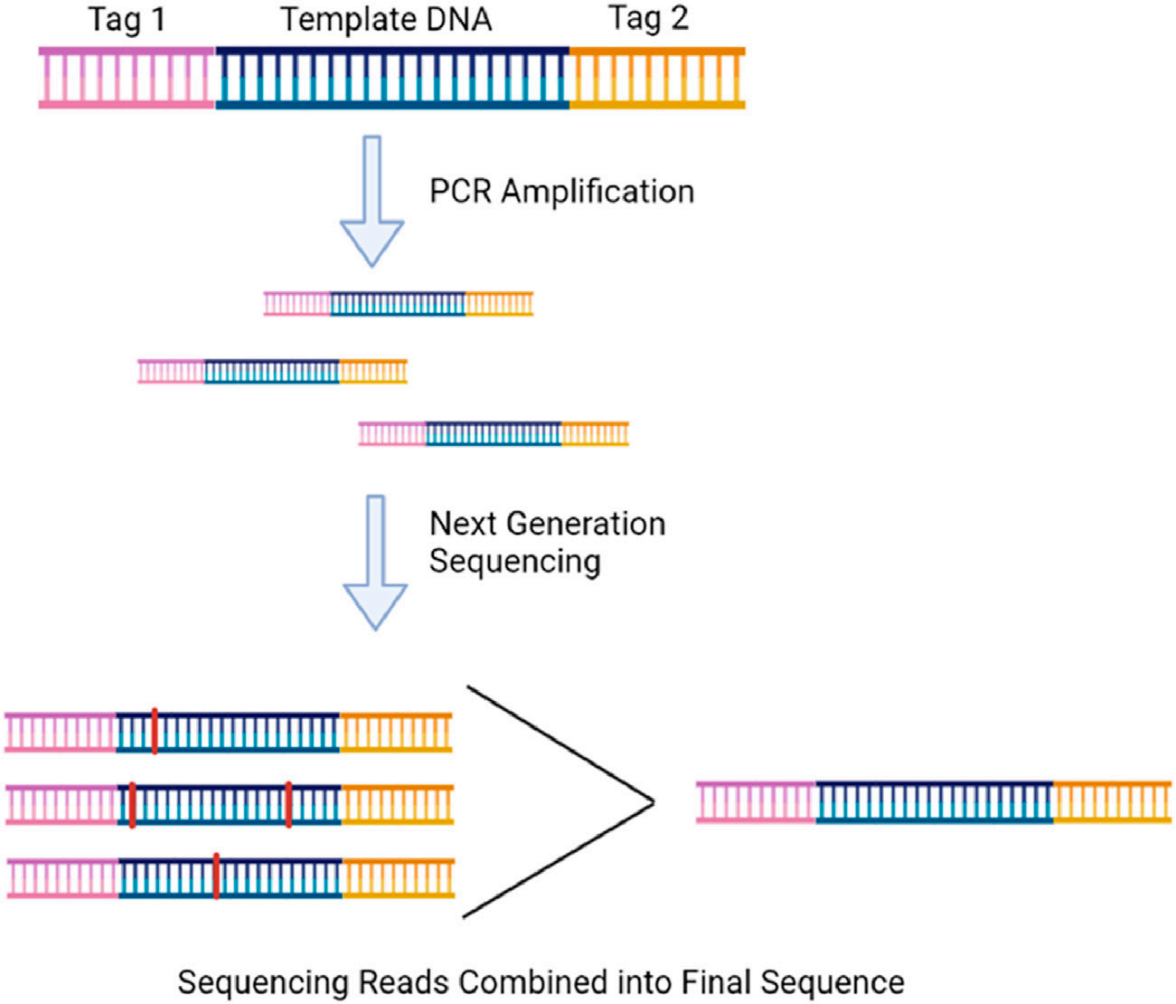
Duplex sequencing involves ligating randomized tags onto the end of every DNA fragment before PCR so that the sequencing reads of the amplified fragments can be compared and a correct consensus sequence can be determined free of PCR-induced error. The illustration is created using BioRender.

Although PCR reactions can confirm the absence of mtDNA deletions, only sequencing can confirm exact sequence changes. Next-generation sequencing (NGS) can routinely sequence many whole mtDNA fragments in parallel to detecting low-level heteroplasmies while being more accurate than its Sanger sequencing predecessor (Gao et al., 2016). It also has a greater throughput than PCR methods when evaluating a greater number of targets (Cheng et al., 2019). Depending on the technology used, however, incorrect base calls can occur between 0.1%–10% of the time (Valentine et al., 2020). Consensus-based error correction is one method to correct incorrect base calling. Duplex sequencing is the most popular version and works as shown in Figure 7. It can reduce the error rate to ∼2 × 10^−8^ and has been extensively used to study somatic mtDNA deletions (Arbeithuber et al., 2020; Abascal et al., 2021).

NGS is typically performed on DNA extracted from thousands of cells at once. Bulk analysis lowers sensitivity, preventing the determination of levels of heteroplasmy in individual cells. This makes many methods incapable of distinguishing between a tissue with a few highly mutant mitochondria from a tissue with the mutation present across more cells at a lower level. Bulk analysis of mtDNA has also been used to construct human cell lineages to determine individual cells’ fates (Ludwig et al., 2019; Lareau et al., 2021). If used to investigate aging, such methods would likely prove useful in better understanding the cellular and physiological impact of mtDNA deletions.

To sequence mtDNA in smaller samples, such as individual cells, mtDNA is often amplified in advance by some form of PCR before NGS is used. This can cause NGS methods to encounter the same limitations introduced by PCR amplification, such as the introduction of mutations. However, the methods are improving. Amplification-free sequence enrichment of whole mtDNA molecules has been achieved by using Cas9 systems with guide RNA-targeting mtDNA. This system has been used in conjunction with full-length nanopore sequencing to localize age-induced mtDNA structural variants, including deletions in the heteroplasmic mtDNA of multiple different types of human cells (Vandiver et al., 2022).

Ultimately, all forms of PCR and sequencing are beneficial in some forms of investigations, be it to quantify shorter deleted mtDNA fragments or to identify exact deletion sequences. To fully determine the extent and distribution of mtDNA deletions between tissues and models through age, various methods should be used in tandem. Yet, most studies discussed use a single method to quantify the mtDNA deletion load. This further complicates the quantitative comparisons between studies and the overall picture of mtDNA deletion’s influence on aging.

## 4 Conclusion

Deletions to mtDNA have a complex association with the aging. While there has been no indisputable link of causation, animal and human models have shown that there is a correlation between the development, pathogenicity, and phenotypes of aging and aging-related diseases. Various factors complicate our ability to investigate and understand the effects of deletions on aging, such as tissue-specific differences, and the interconnectedness of all metabolic systems and their aging phenotypes. Because evidence for and against mtDNA deletions as a driver of aging is conflicted, further investigation into the impacts in different model organisms and biological pathways is crucial to establish or exonerate their level of influence in aging.

Currently, there is no complete and inexpensive quantification method in which the entire mtDNA molecule of multiple cells can be assessed for deletions independently of one another. Additionally, the majority of mtDNA studies focus on the quantification and effects of point mutations. Therefore, there is a need for the continued development of PCR assays with greater throughput, sensitivity, and ability to assess greater numbers of targets across the mitochondrial genome.

## Abbreviations

ETC, electron transport chain; mtDNA, mitochondrial DNA; mtDNA4977, mitochondrial DNA4977 common deletion; OXPHOS, oxidative phosphorylation; ROS, reactive oxygen species.
